# Citations for Randomized Controlled Trials in Sepsis Literature: The Halo Effect Caused by Journal Impact Factor

**DOI:** 10.1371/journal.pone.0169398

**Published:** 2017-01-03

**Authors:** Zhongheng Zhang, Sven Van Poucke

**Affiliations:** 1 Department of emergency medicine, Sir Run-Run Shaw Hospital, Zhejiang University, School of Medicine, Hangzhou, P.R. China; 2 Department of Anesthesia, Critical Care, Emergency Medicine and Pain Therapy, Ziekenhuis Oost-Limburg, Genk, Belgium; University of Illinois-Chicago, UNITED STATES

## Abstract

Citations for randomized controlled trials (RCT) are important for the dissemination of study results. However, predictors of citations for RCTs have not been investigated. The study aimed to investigate the predictors of citations for RCTs in sepsis literature. RCTs that investigated the efficacy of treatment strategies on clinical outcomes in sepsis patients were included, and publication dates were restricted to the period from 2000 to 2016. Risk of bias was assessed using the Cochrane handbook for systematic reviews and interventions. A multivariable linear regression model was built to investigate the independent variables associated with total citations. In total, 160 RCTs met our inclusion criteria and were included for analysis. The median of total citations was 28.5 (IQR: 6–76). The journal impact factor (IF) for articles was 6.312 (IQR: 3.143–7.214). The dependent variable was transformed by the square root to improve normality and meet the assumption of homoscedasticity. The journal IF (coefficient: 0.2; 95% CI: 0.16, 0.25) was independently associated with total citations. Large samples were associated with more total citations (coefficient: 0.0026; 95% CI: 0.0013, 0.0039). The study demonstrated that the journal IF was a major determinant of the RCT’s total citation number.

## Introduction

Randomized controlled trials (RCT) are fundamental to provide high-quality evidence for clinical practice. Therefore, the dissemination of RCT results is of crucial interest for authors, editors and readers. Publication in peer-reviewed journals is a major approach to disseminate the result of RCT findings. Metrics to quantify dissemination of an RCT include the number of reads and downloads from the website, which might be inaccurate. The number of citations for an article provides a much more accurate quantification of dissemination, considering that authors carefully select their reference lists and only the most important work is cited (e.g., some journals limit the number of references).

The publication of RCT should not be considered the end of the entire project. The dissemination of the knowledge is equally important, as is the verification of whether the RCT was up-to-date based on the current state of the art. Therefore, predicting the number of citations for an article is a potential interest to both authors and editors. The journal impact factor (IF) is an important bibliometric variable reflecting the impact of a journal [1–3]. Some studies suggest that IF is a strong predictor of citation, while others report that the quality of study design is equally important [4–6]. However, these studies included all types of original articles and systematic reviews. To the best of our knowledge, no study has investigated the predictors of citations in RCTs. In this study, we focused on RCTs because validated tools were available to assess the risk of bias. In addition, we narrowed our topic to the treatment of sepsis, making the study more homogeneous.

## Methods

### Study identification

RCTs comparing the effectiveness of different treatments on clinical outcomes were included. The subjects were patients who had sepsis at randomization. The exclusion criteria included 1) animal experiments; 2) septic arthritis; 3) duplicates or a secondary analysis of original trials; 4) non-randomized trials; 5) studies involving non-sepsis or prevention of sepsis; 6) study protocols; 7) systematic reviews; and 8) educational training about sepsis management.

The ISI Web of Science was searched for relevant articles. The searching strategy consisted of key terms of sepsis and randomized controlled trials. We restricted the publication dates from 2000 to May 2016 (the time when the study was conducted). The study was performed according to the PRISMA 2009 checklist (S1 File)

### Data extraction

Data were extracted at the journal and article levels. The journal information included IF and journal title. The journal IF is calculated by dividing the number of current year citations to the source items published in that journal during the previous two years. In this study, we used the 2015 IF reported by the journal citation report (JCR). The article information included the article title, the number of participating centers, sample size, the sample size calculation, the results (negative, positive or neutral), whether an academic study group was involved in the study, publication year, doi, whether the study was presented at conferences, the total citations, the average citations per year, and the individual year citation numbers from 2000 to 2016. Only the primary outcome was used to assess whether the study result was positive, negative or neutral. If the primary outcome was not explicitly specified, the conclusion in the abstract section was assessed to determine the result.

### Risk of bias

The risk of bias was assessed according to the Cochrane handbook for systematic reviews and interventions [7]. The sequence generation was judged as low risk when the authors described a random component of sequence generation such as random number table, computer random number generator, coin tossing, and throwing dice. Allocation concealment was adequate when the authors described central allocation, sequentially numbered drug containers of identical appearance, and sequentially numbered, opaque, sealed envelopes. Blinding was at low risk of bias when the description was clear or the outcome measurement was unlikely to be influenced by non-blinding. Incomplete outcome data was determined as low risk when there was no missing outcome or when the reason for missing outcome was unlikely to relate to the true outcome. Selective reporting was considered to be high risk when pre-specified outcomes were not reported.

### Statistical analysis

Data were expressed as medians (interquartile range) or means (standard deviation) for continuous variables as appropriate [8]. Included RCTs were separated into highly cited and poorly cited groups using the median total citation number as the cutoff. Student’s t-test was used to compare normally distributed data, and Wilcoxon rank sum test was used for skewed data. Categorical variables were expressed as numbers and percentages, and they were compared using Chi-square tests. We assumed that total citations were associated with journal IF and publication year. Total citations were plotted against IF, stratified by publication year. Included RCTs were divided into two groups by the median of total citations. Characteristics were compared between the two groups. Multivariable linear regression model was built to explore explanatory variables that were associated with total citations. Before model fitting, associations among continuous variables were explored using means in a scatter matrix. Because the number of participating centers and the sample sizes were closely associated with each other, only sample size was incorporated into the model to avoid collinearity. As such, the full model included the following variables: sequence generation, allocation concealment, blinding of participants, blinding of outcome assessors, incomplete outcome data, selective reporting, sample size calculation, sample size, results, impact factor, cooperation group, conference, and publication year. After fitting the model, homoscedasticity was examined using a scale-location graph. There was a correlation between fitted values and square roots of standardized residuals. Furthermore, the Q-Q plot demonstrated that the normality assumption was not satisfied. The total citations were transformed by square root, and the model was refit. Stepwise backward elimination and forward selection using the Akaike information criterion was employed to select important variables [9]. All statistical analyses were performed using R (version 3.2.3), and a two-tailed p-value less than 0.05 was considered to be statistical significance.

## Results

**Fig 1:**
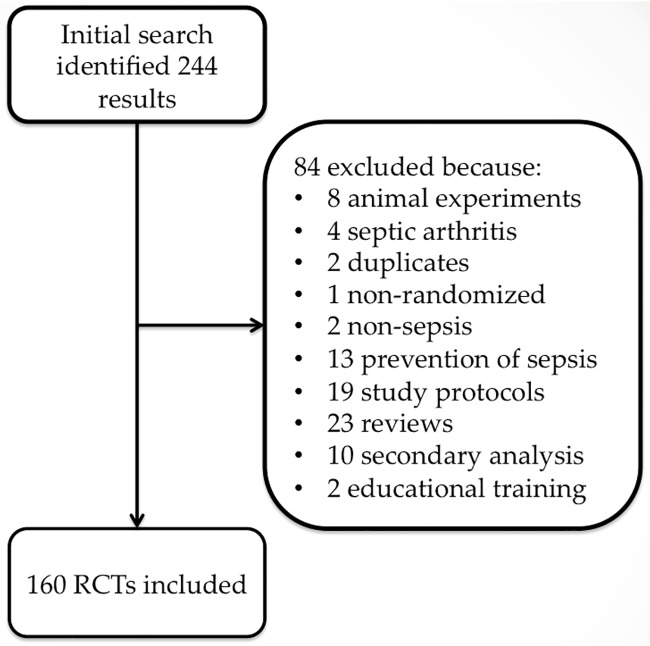
Flow chart of study selection.

The initial search identified 244 articles. Full texts of these articles were screened manually for potential eligible studies. Eighty-four articles were excluded because there were 8 animal experiments, 4 septic arthritis studies, 2 duplicates, 10 secondary analyses of original trials, 1 non-randomized trial, 2 studies involving non-sepsis, 13 investigations of the prevention of sepsis, 19 study protocols, 23 systematic reviews, and 2 educational training documents on sepsis management. In total, 160 RCTs met our inclusion criteria and were included for analysis (Fig 1).

**Fig 2:**
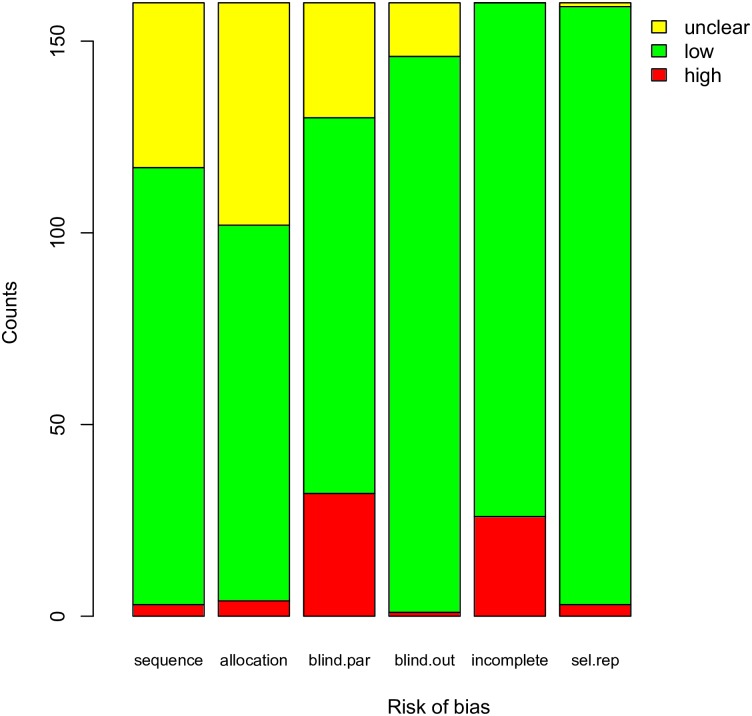
Summary of risk of bias for the included randomized controlled trials.

Fig 2 displays the risk of bias for each item. Sequence generation was performed well in most RCTs. Allocation concealment was performed less well, and 58 RCTs did not explicitly describe the method for allocation concealment. Blinding of participants was denoted as “high risk” in 32 RCTs. Because most RCTs employed a solid outcome variable such as mortality and some laboratory measurements, the assessment of outcome was less likely to be influenced by blinding. Thus, 145 RCTs (91%) were considered to have a low risk of bias. Twenty-six RCTs were considered to have a high risk of attrition bias because they reported a significant number of losses of follow-up. Selective reporting was at low risk of bias for most trials.

The characteristics of included RCTs are summarized in Table 1. Sample size calculations were performed in 105 (65.5%) RCTs. The median sample size was 90 (interquartile range [IQR]: 48–256). The median number of participating centers was 1 (IQR: 1–11). Most studies reported neutral results (97, 60.6%), followed by positive results (56, 35%) and negative results (7, 4.4%). Forty RCTs were performed by academic study groups. The median publication year was 2010 (IQR: 2006–2014). Five trials were explicitly reported as having been presented during a conference. The median of total citations was 28.5 (IQR: 6–76). Average citations per year for an RCT was 4.36 (IQR: 1.67–7.9). The journal IF for articles was 6.312 (IQR: 3.143–7.214). RCT characteristics were compared between highly and poorly cited groups. These findings showed that there were significantly more participating centers in the highly cited group compared with the poorly cited group (3 [1, 22.3] vs. 1 (1, 6); p = 0.011). RCTs in the highly cited group were published earlier than those in the poorly cited group (2007 (2003, 2010) vs. 2014 (2012, 2015); p<0.001). Journal IFs of highly cited RCTs were significantly greater than those in poorly cited RCTs (6.312 (5.473, 13) vs. 4.442 (1.856, 6.312); p<0.001).

**Table 1:** Comparisons of characteristics between highly and poorly cited RCTs.

Characteristics	Overall	Poorly-cited (≤28.5 times, n = 80)	Highly cited (>28.5 times, n = 80)	p-value
Sequence generation				0.1716
High	3 (1.9)	0 (0)	3 (3.8)	
Low	114 (71.3)	60 (75)	54 (67.5)	
Unclear	43 (26.8)	20 (25)	23 (28.8)	
Allocation concealment				0.54
High	4 (2.5)	1 (1.2)	3 (3.8)	
Low	98 (61.3)	51 (63.8)	47 (58.8)	
Unclear	58 (36.2)	28 (35)	30 (37.5)	
Blinding of participants				0.2483
High	32 (20)	16 (20)	16 (20)	
Low	98 (61.3)	45 (56.2)	53 (66.2)	
Unclear	30 (18.7)	19 (23.8)	11 (13.8)	
Blinding of outcome assessor				0.524
High	1 (0.6)	0	1 (1.2)	
Low	145 (90.6)	72 (90)	73 (91.3)	
Unclear	14 (8.8)	8 (10)	6 (7.5)	
Incomplete outcome				0.5203
High	26 (16.3)	15 (18.8)	11 (13.8)	
Low	134 (83.7)	65 (81.2)	69 (86.2)	
Selective reporting				0.1286
High	3 (1.9)	0	3 (3.8)	
Low	156 (97.5)	80 (100)	76 (95)	
Unclear	1 (0.6)	0	1 (1.2)	
Sample size calculation (n, %)	105 (65.6)	55 (68.8)	50 (62.5)	0.5055
Sample size (median, IQR)	90 (48, 256)	73.5 (48.6, 180)	104.5 (44.8, 303)	0.2153
Participating centers (median, IQR)	1 (1,11)	1 (1, 6)	3 (1, 22.3)	0.011
Results (n, %)				0.3541
Positive	56 (35)	26 (32.5)	30 (37.5)	
Neutral	97 (60.6)	52 (65)	45 (56.3)	
Negative	7 (4.4)	2 (2.5)	5 (6.2)	
Academic cooperation group (n, %)	40 (25)	15 (18.7)	25 (31.3)	0.1003
Publication year (median, IQR)	2010 (2006, 2014)	2014 (2012, 2015)	2007 (2003, 2010)	<0.001
Presentation to a conference (n, %)	5 (3.1)	0	5 (6.3)	0.07
Total citations (median, IQR)	28.5 (6,76)	6 (1, 18.3)	76 (47.8, 132.2)	<0.001
Average citations per year (median, IQR)	4.36 (1.67, 7.90)	1.67 (0.5, 7.90)	8.14 (5.21, 13.83)	<0.001
Impact factor (median, IQR)	6.312 (3.143, 7.214)	4.442 (1.856, 6.312)	6.312 (5.473, 13)	<0.001

**Fig 3:**
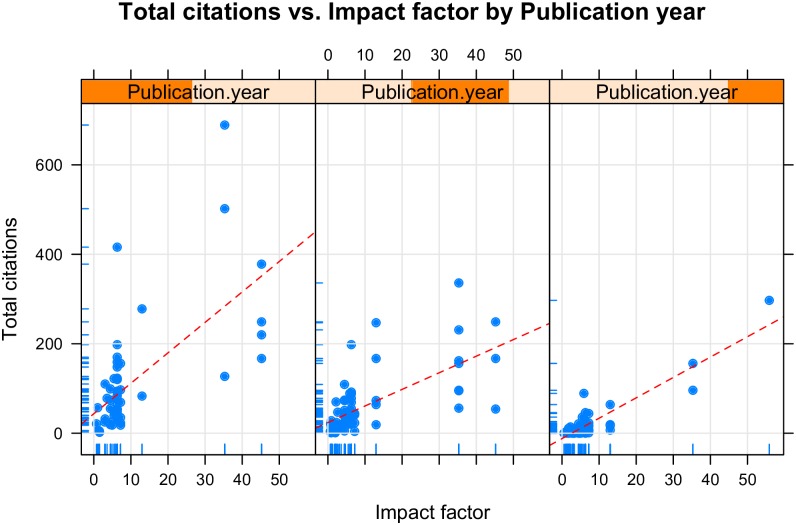
Scatter plots of the total citations against the journal impact factor, stratified by publication year.

**Fig 4:**
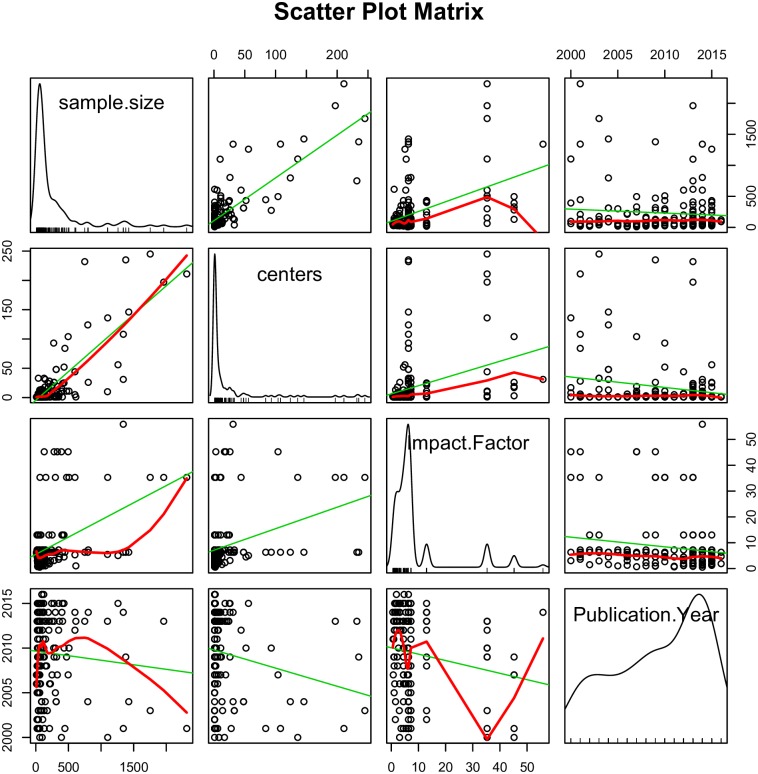
Scatterplot matrix describing the correlations among continuous variables. Participating centers and sample sizes appeared to be closely correlated with each other, thus we only retained sample size in the model.

**Fig 5:**
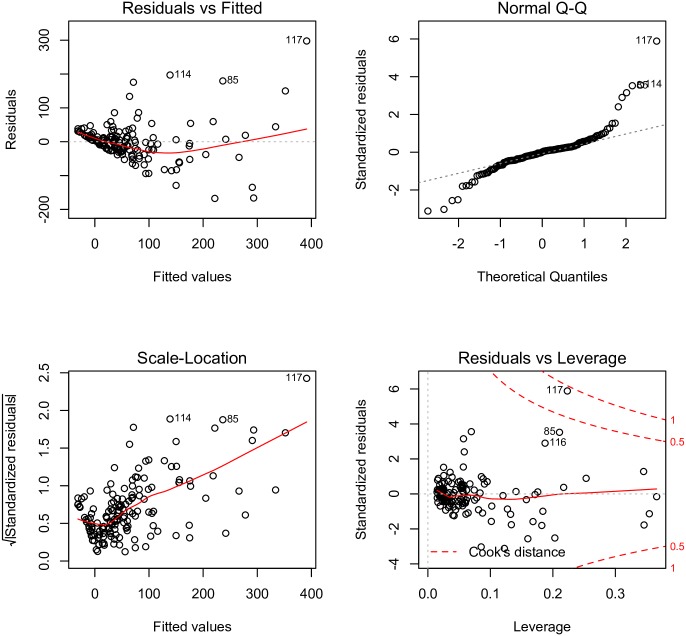
Diagnostic plots of multivariable linear regression. The dependent variable, total citation, was not transformed. The scale-location plot shows a correlation trend between fitted values and square root of the standardized residual, indicating the violation of homoscedasticity. The Q-Q plot also shows the violation of normality.

In Fig 3, publication year was divided into three categories, with each category containing an equal number of RCTs. The total number of citations clearly increased with journal IF and publication year. However, there are large residuals that cannot be fully explained by the two variables. To avoid collinearity among independent variables, all continuous variables were plotted against each other (Fig 4). Participating centers and sample sizes appeared to be closely correlated to each other, thus we only retained sample size in the model. Initially, we fit the model without transforming the dependent variable (total citation). The model assumptions were examined with plots (Fig 5). The scale-location plot shows a correlation trend between fitted and square root of standardized residual, indicating a violation of homoscedasticity. Additionally, the Q-Q plot shows the violation of normality. Therefore, square root transformation was applied for the dependent variable [10].

**Fig 6:**
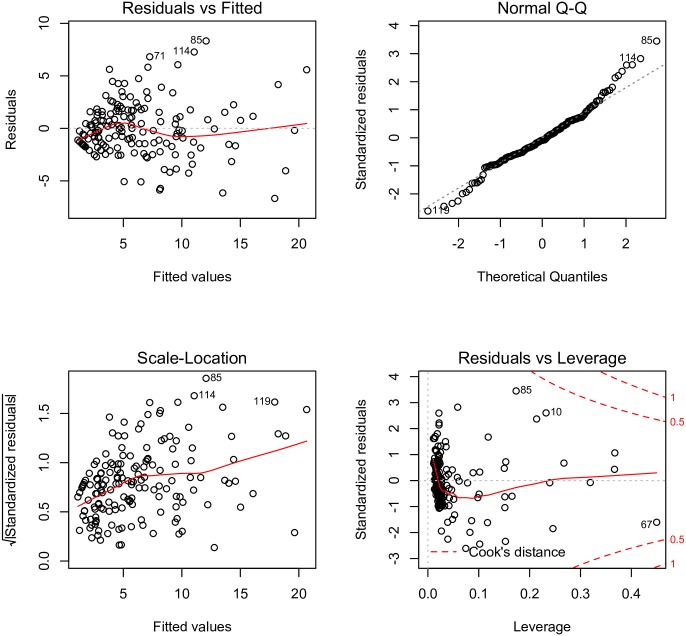
Diagnostic plots of multivariable linear regression with the dependent variable total citation transformed by square root. The relationship between the fitted values and square root of standardized residual was weakened. The Q-Q plot showed that the normal assumption of the dependent variable was well satisfied.

The final model was built using a stepwise approach (Table 2). Coefficients reported here were adjusted for other factors (e.g., holding all other factors constant). The journal IF (coefficient: 0.2; 95% CI: 0.16, 0.25) and publication year (coefficient: -0.48; 95% CI: -0.57, -0.39) were independently associated with the total citation. Because earlier papers had more time to accumulate citations, it was not surprising that publication year was a significant contributing factor. The risk of bias in the original RCT was not significantly associated with total number of citations, except for selective reporting. RCTs with a low risk of bias had fewer total citations than those with a high risk of bias (coefficient: -4.46; 95% CI: -7.65, -1.27). Sample size was positively correlated with the number of total citations (coefficient: 0.0026; 95% CI: 0.0013, 0.0039). RCTs with neutral results were less likely to be cited than those with negative results (coefficient: -2.73; 95% CI: -4.84, -0.26). Conference presentations tended to improve the number of total citations (coefficient: 1.97; 95% CI: -0.54, 4.49). The model showed a moderate fit to the data, accounting for 70% of total variance. The diagnostic plots showed that model assumptions were satisfied in the model with transformed dependent variables. The Q-Q plot showed that the normal assumption of the dependent variable is well satisfied (Fig 6).

**Table 2:** Linear model built by a stepwise approach, with a square root transformation of the total citation outcome variable.

	Coefficient	Lower limit of 95% CI	Upper limit of 95% CI	p
(Intercept)	967.53	785.54	1149.53	<0.001
Selective reporting (low vs. high)	-4.46	-7.65	-1.27	0.006
Selective reporting (unclear vs. high)	-5.03	-11.17	1.12	0.108
Sample size	0.0026	0.0013	0.0039	<0.001
Outcome (neutral vs. negative)	-2.73	-4.84	-0.62	0.012
Outcome (positive vs. negative)	-1.94	-4.09	0.21	0.077
Impact factor	0.20	0.16	0.25	<0.001
Conference presentation	1.97	-0.54	4.49	0.123
Publication year	-0.48	-0.57	-0.39	<0.001

Residual standard error: 2.655 on 151 degrees of freedom

Multiple R-squared: 0.7157, Adjusted R-squared: 0.7006

F-statistic: 47.51 on 8 and 151 DF, p-value: < 2.2e-16

Sensitivity analyses were performed by excluding RCTs with citations greater than 500. Multivariable linear regression model was fit (Table 3), which showed similar results. Journal IF was significantly associated with total citations (coefficient: 3.8, CI: 3, 4.5; p<0.001). In this model, the outcome variable was not transformed by the square root, thus the coefficient of journal IF could be interpreted such that each one point increase in journal IF resulted in an increase of 3.8 citation counts.

**Table 3:** Sensitivity analysis by excluding RCTs with total citations greater than 500 without transforming the outcome variable.

	Coefficient	Lower limit of 95% CI	Upper limit of 95% CI	P
(Intercept)	10637.8	7374.7	13900.9	<0.001
Selective reporting (low vs. high)	-89.9	-146.4	-33.5	0.002
Selective reporting (unclear vs. high)	-108.2	-217.1	0.6	0.051
Sample size	0.025	0.0013	0.0039	0.059
Outcome (neutral vs. negative)	-81.4	-118.8	-44.1	<0.001
Outcome (positive vs. negative)	-68.8	-107.0	-30.6	<0.001
Impact factor	3.8	3.0	4.5	<0.001
Conference presentation	26.8	-16.0	73.1	0.207
Publication year	-5.2	-6.8	-3.6	<0.001

Residual standard error: 47.02 on 149 degrees of freedom

Multiple R-squared: 0.6273, Adjusted R-squared: 0.6073

F-statistic: 31.34 on 8 and 149 DF, p-value: < 2.2e-16

## Discussion

The study demonstrated that journal IF was the major determinant of an RCT’s total citation count. As expected, the total number of citations was dependent on the publication year. Early publications showed significantly more total citations. Sample size, which was closely correlated with the number of participating centers, was also independently associated with total number of citations. RCTs with negative results were more likely to be cited than neutral results, but the magnitude was not large. In addition, conference presentations tended to promote dissemination of RCT results and increase its total number of citations.

Although predictors of citations to RCT have never previously been investigated, several studies have explored predictors of citations to journal articles. Consistent with our findings, journal IF of an article was found to be the most important determinant of the number of citations to that article [11]. These articles included retrospective and prospective trials. If the contribution of IF to a citation was rated as 100, the contributions of sample size, Delphi score, presentation at meeting and study design (retrospective vs. prospective) were 26.5, 26, 5.5 and 2.7, respectively. Positive results had no contribution to the citations [12]. By using multiple regression analysis, Pamela Royle and colleagues found that journal IF explained over half of the variation in citations to systematic reviews [4]. Correlation analysis showed that the 2-year IF on Web of Science was significantly associated with number of citations (Rho = 0.259), but the IF was not incorporated into subsequent multivariable analysis [13]. This study involved systematic reviews in skin diseases. Journal IF was associated with total citations to an RCT in the area of sepsis, independently from risk of bias, sample size, participating centers and other relevant trial-level characteristics. Journal IF may have a halo effect on subsequent citations [14,15]. If there are several RCTs of comparable study design and risk of bias, investigators tend to cite RCTs that are published in high IF journals. The halo effect is defined as a cognitive bias in which an observer's overall impression of an object influences the observer's feelings and thoughts about that entity's character or properties [16]. The impact of an IF on RCT’s total citations can be regarded as a halo effect in bibliometrics.

It is surprising that the risk of bias of RCT had no impact on total citations. In other words, the authors generally do not consider the quality of evidence when citing an article. This finding is inconsistent with other findings. In orthopedic literature, the study design was found to be the only variable associated with the subsequent citation rate. RCTs, meta-analyses and basic science papers had significantly more citations (mean 15.5, 9.3 and 7.6, respectively) than observational studies (mean retrospective 5.3, prospective 4.2) and case reports (mean 1.5) (p = 0.01). However, this study investigated articles published in the journal with the highest IF in orthopedics, and journal IF was not incorporated into the analysis [5]. Furthermore, this study included all types of study designs such as RCTs, retrospective designs, animal studies and systematic reviews. Researchers may have more awareness for the impact of study designs on final conclusions than the impact of risk of bias of RCTs. Furthermore, the risk of bias is usually not explicitly highlighted in an RCT article, and quality of reporting is always very poor [17–19].

Conference presentations of scientific work have been noted to help disseminate its findings [20]. Our study suggested a trend towards more total citations for articles that are also presented to a conference. The sample size of our study was probably not large enough to have the statistical power to detect this difference. Previous studies have also found that the group authorship was associated with more citations (coefficients: 11.1; 95% CI: 2.7, 19.5) [21]. This association was not identified in our multivariable analysis. Of note, data on conference presentations were extracted from the ISI web of science, which may not be exhaustive. Only five of the 160 RCTs were identified as having been presented at a conference.

The strength of the study is the robust methodology in building the regression model. There is evidence that count variables, such as the number of citations, were usually not normally distributed and the assumption of homoscedasticity is usually violated. Previous studies in this field have failed to address this problem, and the results may be biased. This problem was well addressed in the present study by transforming the dependent variable with square root. The study restricted to RCTs, whose results can be more homogenous. Previous studies incorporated all types of articles, including animal studies, retrospective studies and prospective trials [5,13,22]. In this situation, the within-study type random effects were not considered, leaving a large portion of unexplained residuals. However, several limitations of the study should be acknowledged. The study involved articles indexed in ISI web of science. Whether our results can be extrapolated to other databases that provide a citation service, such as Scopus and Google scholar, is largely unknown [23]. Our model is only moderately fitted, accounting for approximately 70% of the variance of the total citations. Some factors, such as the number of authors, article length, and open access status, may be potential predictors of citations [6,24,25].

## Supporting Information

S1 File PRISMA 2009 checklist. (DOC)
